# Development and Validation of a Multimodal Emotion Recognition Ability Test Based on the Chinese Cultural Context

**DOI:** 10.3390/jintelligence14080169

**Published:** 2026-08-01

**Authors:** Xiaoming Song, Jinmei Leng, Xia Wu, Sheng Yang, Fang Luo

**Affiliations:** 1 Faculty of Psychology, Beijing Normal University, 19 Xinjiekouwai Street, Haidian District, Beijing 100875, China; 2 School of Computer Science and Technology, Beijing Institute of Technology, Beijing 100081, China; 3 Li Auto Inc., 11 Wenliang Street, Shunyi District, Beijing 101399, China

**Keywords:** emotion recognition ability, multimodal database, test development, category recognition, intensity recognition

## Abstract

Emotion recognition is essential for social adaptation and mental health. However, existing measures still rely on limited stimulus materials or simplified task formats, which restricts ecological validity. The present study aimed to construct a localized multimodal emotion expression database in the Chinese context and to develop a multimodal emotion recognition ability test integrating emotion category and intensity recognition. In Study 1, a multimodal emotion expression database was established through actor performances, expert evaluation, and participant validation. The final database contained 2160 videos representing six basic emotions, five levels of emotional intensity, and two expression modes (vocal and non-vocal). In Study 2, 90 videos were selected under three recognition conditions (vocal-expression, muted vocal-expression, and muted non-vocal-expression). An integrated scoring approach combining emotion category and intensity was used to develop the test. A total of 236 valid participants completed the test. The results indicated that, after 61 video clips were retained, the test demonstrated good reliability and item discrimination. Evidence for criterion-related validity and construct validity was also obtained. Overall, the findings suggest that the present measure is a reliable and valid tool for assessing multimodal emotion recognition ability in the Chinese cultural context and offers the first empirical evidence that integrating category and intensity recognition can better capture the multidimensional nature of this ability.

## 1. Introduction

Emotion recognition ability is widely regarded as a core component of emotional intelligence (Connolly et al., 2020). It refers to the capacity to infer others’ emotional states from nonverbal cues such as facial expressions, body posture, and vocal intonation (Bänziger et al., 2009; Mayer et al., 2016). It is closely linked to social adaptation and psychological well-being. Individuals with higher emotion recognition ability are better able to interpret others’ emotional signals and adjust their responses accordingly (Philippot & Feldman, 1990; Sommer & Schlegel, 2025), which facilitates smoother social interactions and more effective adaptation. In contrast, deficits in emotion recognition are commonly observed in various psychological disorders, including autism and schizophrenia (Fenske et al., 2015; Joormann & Gotlib, 2006; Surguladze et al., 2004). These deficits may further undermine mental health by impairing interpersonal functioning and reducing perceived social support. Therefore, accurate emotion recognition not only supports the development of high-quality social relationships but may also promote mental health through enhanced social support (Sommer et al., 2025). Given the growing prevalence of social difficulties and mental health problems in contemporary society, developing high-quality measures of emotion recognition ability could provide reliable support for the early screening of psychological disorders and the evaluation of intervention effectiveness.

In naturalistic contexts, emotional information is communicated through multiple channels, such as facial expressions, body movements, and vocal signals. Accurate recognition of others’ emotional states therefore requires the dynamic integration of information across these channels (Tsiourti et al., 2019). Compared with unimodal tests, multimodal assessments are better suited to capturing this integrative process and thus provide a more ecologically valid representation of emotion recognition in everyday life. However, many existing “multimodal” tests simply combine cues from different unimodal test (Globerson et al., 2015; Martini et al., 2023) and rely mainly on vocal expression (Bänziger et al., 2012), limiting their ability to capture cross-modal integration in real-world emotion recognition and making it difficult to reflect individuals’ ability to generalize across different expressive contexts. At the same time, many tests use stimuli drawn from Western databases (Yu et al., 2022), even though emotional expression and recognition are strongly shaped by cultural background. Previous research has shown that in terms of emotional expression, Westerners tend to favor overt emotional expression, while Easterners are more subtle and restrained in their emotional expression (Kraus et al., 2024), relying primarily on eye contact rather than facial muscle movements. Individuals exhibit a significant in-group advantage in recognizing emotional expressions (Elfenbein & Ambady, 2002; Liu et al., 2022), meaning that they are more accurate at recognizing emotional expressions from their own cultural group. Therefore, the lower accuracy of Eastern participants in recognizing Western faces is not due to lower emotion recognition ability per se, but rather to cross-cultural decoding bias. This may compromise the validity of the test to some extent.

Another important limitation lies in the assessment of emotional intensity. Emotional intensity is a crucial dimension of emotional information. It not only shapes how individuals understand others’ emotional states, but also influences the selection of appropriate emotion regulation strategies, yet it remains insufficiently addressed in existing tests. Assessments that focus only on emotion categories while neglecting intensity may fail to fully capture emotion recognition ability in real-world contexts. Moreover, intensity scales vary considerably across studies: some are too coarse to detect subtle individual differences, whereas others are so fine-grained that they may increase noise and reduce measurement stability (Bänziger et al., 2012; El Zein et al., 2018; Ito et al., 2017; Yau et al., 2020).

To address the limitations of existing tests in cross-modal information representation, culturally inappropriate materials, and insufficient attention to emotion intensity recognition, the present study developed an emotion recognition test adapted to the Chinese cultural context through two sub-studies. Study 1 aimed to construct a localized multimodal emotion expression database featuring Chinese actors as expressers. The materials were designed to include five intensity levels and three recognition conditions (vocal-expression, muted vocal-expression, and muted non-vocal-expression), so as to better approximate real-life emotion recognition situations. Subsequently, we examined whether the video materials could effectively convey the intended emotion categories and intensity levels. Study 2 aimed to develop and validate a multimodal emotion recognition ability test based on this database. Through these two sub-studies, the present study aimed to provide a more ecologically valid and culturally appropriate tool for assessing multimodal emotion recognition ability, with the potential to inform both basic research on emotion processing and applied assessment in clinical and educational settings.

### 1.1. Multi-Modal Emotion Recognition

Emotion recognition ability refers to an individual’s capacity to accurately discern emotional information from nonverbal cues, including facial expressions, body posture, and vocal intonation (Bänziger et al., 2009; Mayer et al., 2016). Early research on emotion recognition primarily adopted a unimodal approach, examining different forms or modalities of emotional expression independently, such as facial expressions or vocal intonation. However, in everyday life, emotional expression is more effectively conveyed through the dynamic interplay of multiple cues, including facial, vocal, and bodily signals (Bänziger et al., 2012). Consequently, research focusing exclusively on a single modality is insufficient to comprehensively and reliably assess an individual’s emotion recognition ability.

Multimodal emotion recognition refers to an individual’s ability to infer others’ emotions by integrating emotional information from multiple channels, such as facial expressions, body posture, and vocal intonation (Bowers et al., 1998; de Gelder & Vroomen, 2000). By contrast, relying on a single modality—such as visual or auditory information—to infer emotion can substantially reduce recognition accuracy because the available information is often incomplete or ambiguous (Wu et al., 2025). Therefore, compared with unimodal emotion recognition, multimodal emotion recognition provides a more comprehensive assessment of an individual’s emotion recognition ability. At present, multimodal emotion recognition tests are widely used in emotion-related research. They have been applied to assess emotion recognition ability among employees, elementary school students, and individuals with mental disorders, and to examine its associations with job performance, social relationships, and other important outcomes (Doucet et al., 2016; Globerson et al., 2015; Parrish et al., 2022; Wang et al., 2019).

### 1.2. Basic Emotion Theory and Dimensional Emotion Theory

The theoretical foundations of existing emotion recognition tests primarily fall into two categories: basic emotion theory (BET) and dimensional emotion theory. BET posits that emotions are evolutionarily shaped, discrete, and functionally specific responses, typically organized into six basic emotion categories. In emotion recognition tests developed on the basis of this theory, participants are required to select the most appropriate basic emotion category from a set of predefined options on the basis of the presented emotional stimuli, such as facial images, audio clips, or videos. By classifying emotions into discrete categories, researchers can efficiently construct emotional stimuli, and the scoring procedure is relatively objective and straightforward. However, emotional experience in real life is considerably richer and more fine-grained. For example, anger may be expressed as irritation, frustration, or rage. Tests developed on the basis of BET have difficulty differentiating such nuances.

Unlike basic emotion theory, the dimensional theory of emotion posits that emotions can be characterized and understood in terms of several latent and continuous dimensions (Russell, 1980; Watson & Tellegen, 1985). Assessments developed on the basis of affective dimensional theory typically require participants to rate emotions continuously along specific dimensions, such as valence and intensity. This approach allows for a more nuanced assessment of multiple emotional dimensions and is well suited to capturing emotional continuity, variations in intensity, and complex or mixed emotional states. However, it may have difficulty distinguishing between emotions that are proximate in dimensional space but differ substantially in subjective experience, such as fear and anger, which are both characterized by high arousal and negative valence yet involve markedly different experiential qualities.

It is important to note that these two major theoretical perspectives are not mutually exclusive; rather, they can be viewed as complementary. Each discrete emotion may be understood as a configuration of specific emotional dimensions (Gu et al., 2019). For example, anger may be characterized by negative valence, high arousal, and approach motivation, whereas fear may be characterized by negative valence, high arousal, and avoidance motivation. Cowen and Keltner (2017) further suggested that emotional experiences exhibit both discrete clustering patterns and continuous variation across multiple dimensions. Therefore, emotion recognition tests should incorporate the assessment of both emotion categories and emotional dimensions.

### 1.3. Existing Emotion Recognition Ability Measures and Their Limitations

Currently, most multimodal emotion recognition tests assess participants’ emotion recognition ability primarily through tasks requiring judgments of emotion categories (Surguladze et al., 2004; Yu et al., 2022). Although LaPalme et al. (2023) included ratings of emotion intensity, these ratings were used only as a criterion for screening test materials and were not incorporated into the formal response tasks. These tests, while operationally simple and readily quantifiable, overlook the important role of emotional intensity in emotion recognition. Research has shown that high-intensity emotional expressions are generally easier to identify than low-intensity expressions (Ekman, 2003; Shimizu et al., 2024). Moreover, individuals appear to rely on distinct encoding mechanisms for emotional intensity when recognizing facial expressions (Chen et al., 2024). Taken together, these findings suggest that sensitivity to emotional intensity constitutes an important component of emotion recognition ability. Accordingly, assessments of this ability should take emotional intensity into account alongside the identification of emotion categories. Existing tests typically construct intensity gradients using stimuli of moderate intensity or above (Bänziger et al., 2012). Although this approach strengthens emotional signals, facilitates identification, and reduces floor effects, it offers limited discriminative power for individuals with relatively strong emotion recognition ability. By contrast, some studies have used stimuli with broader intensity intervals, such as 30%, 50%, and 70% intensity levels (El Zein et al., 2018; Ito et al., 2017; Yau et al., 2020). This strategy enhances emotional signals and may improve the detection of individual differences. However, in everyday life, emotional expressions vary continuously and are often conveyed through subtle cues. As a result, designs based on widely spaced intensity levels may fail to capture an individual’s recognition ability under conditions of fine-grained intensity variation. In contrast, a five-level intensity-gradient design extends into the low-intensity range, thereby enabling the assessment of individual sensitivity to subtle emotional cues and allowing for more precise differentiation of recognition ability across varying levels of emotional intensity. In this way, it may enhance the ecological validity and representativeness of the test.

In terms of test format, most multimodal emotion recognition assessments rely on audio and visual presentation. Compared with information conveyed through a single channel, multimodal information provides richer emotional cues and allows individuals to capture a more comprehensive range of expressive characteristics. Such formats are also better suited to assessing cross-modal integration ability. For example, some test designs present facial expressions and emotional vocal cues simultaneously and ask participants to judge whether the emotional information is congruent across modalities (Van Rheenen & Rossell, 2014). However, some existing tests still rely on a relatively simple combination of unimodal components, such as presenting facial expressions and emotional voices separately and asking participants to identify the corresponding emotion categories (Globerson et al., 2015; Martini et al., 2023; Monroy et al., 2025). This separation of visual and auditory elements limits the assessment of an individual’s ability to integrate and use multiple emotional cues simultaneously. By contrast, video-based formats allow emotional information from multiple channels to be presented concurrently, enabling individuals to perceive more intuitively the dynamic unfolding of emotional cues over time. This format can therefore enhance both participant engagement and the realism of the assessment context. In addition, video-based assessments can facilitate the efficient generation and presentation of emotional materials across modalities, thereby improving research convenience and efficiency. At present, video is increasingly emerging as a primary format for the assessment of multimodal emotion recognition ability. For example, Laukka et al. (2021) used 24 silent videos and 24 videos with sound from the GEMEP corpus to assess individual differences in emotion recognition ability. Berrios-Martos and Palomera (2025) developed a test of teachers’ emotional intelligence that included 12 teaching-related situational videos. Similarly, Israelashvili and Fischer (2023) developed an Emotional Accuracy Test based on videos depicting spontaneous emotional expressions in naturalistic settings.

Existing tests materials are assembled from Western stimulus databases (Van Rheenen & Rossell, 2014; Yu et al., 2022). Although this approach reduces the time and cost involved in test development, it may also pose risks to the ecological validity of the assessment. Evidence suggests that emotional expressions vary across cultural contexts. In particular, Western individuals tend to display emotions more overtly, whereas Eastern individuals often express emotions in more subtle and restrained ways (Kraus et al., 2024). Such differences may also be reflected in the relative use of different expressive cues, including greater reliance on the eye region rather than broader facial muscle movements. In addition, research has consistently demonstrated an in-group advantage in emotion recognition (Elfenbein & Ambady, 2002; Liu et al., 2022), whereby individuals are generally more accurate at recognizing emotional expressions produced by members of their own cultural group. As a result, Eastern participants may show lower accuracy when decoding emotional expressions presented by Western faces, not necessarily because of lower emotion recognition ability per se, but because of cross-cultural decoding bias. This issue may in turn compromise the validity and fairness of the assessment.

Furthermore, many existing video-based materials predominantly feature vocalized emotional expressions. Although vocalized expressions are dynamic and provide rich emotional information, they do not fully capture the range of emotional interactions encountered in everyday life, where emotions are also conveyed in silent or minimally verbal contexts. As Berry et al. (2022) and Favelle et al. (2026) noted, emotional communication in naturalistic settings involves interactions among multiple channels, and body movements and postures often amplify or reinforce emotional information conveyed by the face and voice. Moreover, when emotions are expressed without vocalization, bodily and facial cues may differ from those observed in vocalized expressions (Kamiloğlu et al., 2021). Incorporating silent emotional expressions into a test may therefore provide a more comprehensive simulation of emotional information in real social situations and allow for a more accurate assessment of emotion recognition ability.

In summary, these considerations suggest the need to develop a new set of video-based test materials that are appropriate for the Chinese cultural context. Such materials would likely improve both the ecological validity and the cultural fairness of the assessment.

### 1.4. The Present Research

Across two studies, we constructed and validated a multimodal emotion recognition ability test suitable for the Chinese cultural context. Study 1 aimed to construct a localized multimodal emotion expression database featuring Chinese actors as expressers, by systematically manipulating emotional intensity (five levels) and information conditions (vocal-expression, muted vocal-expression, and muted non-vocal-expression), in order to address the limitations of existing materials in terms of intensity gradation and cultural appropriateness, thereby filling the gap in dynamically varying emotional stimulus materials within the Chinese cultural context. Study 2 aimed to develop and validate a multimodal emotion recognition test based on this database, by creatively introducing a scoring approach that integrates emotion category recognition and intensity recognition, so as to provide a more comprehensive assessment of individual differences in emotion recognition ability.

## 2. Study 1

In Study 1, we aimed to construct and validate a localized database of dynamic emotional expressions that could serve as a source of high-quality, standardized stimuli for subsequent test development. To address several limitations of existing measures, including insufficient intensity gradation, limited cultural relevance, and reliance on a single informational condition, the present study incorporated a five-level intensity gradient and three recognition information conditions during stimulus construction (vocal-expression, muted vocal-expression, and muted non-vocal-expression). Six professional actors (three men and three women) were recruited for filming, resulting in a total of 2160 video clips representing six basic emotions: happy, sad, angry, disgust, fear, and surprise. Each emotion was portrayed at five levels of intensity, ranging from low to high. By recording both vocalized and non-vocalized expressions, we created materials that captured different recognition conditions and more closely approximated real-world situations in which emotional cues are available across multiple modalities or through a single modality alone. The procedure provided an empirical basis for item selection and test construction in Study 2.

### 2.1. Methods

Drawing on the stimulus-generation procedure of the Geneva Multimodal Emotion Portrayals Core Set (Bänziger et al., 2012), which is grounded in contextually guided naturalistic expression, we developed an audiovisual set of dynamic emotional stimuli. Specifically, the present study employed a three-phase methodological framework for stimulus development and validation. First, emotional scenario texts were generated and pilot-tested to ensure their effectiveness in eliciting the intended emotions at specific intensity levels. Second, professional actors performed these scenarios while their facial, bodily, and vocal expressions were recorded. Third, the resulting video materials were validated through both expert judgments and naïve participant ratings to establish a reliable scoring key and to examine their difficulty distribution.

#### 2.1.1. Emotional Context Text Generation

First, psychological experiences corresponding to five intensity levels (1-mild, 2-moderate, 3-severe, 4-intense, and 5-extreme) were defined for each of the six basic emotions (happy, sad, angry, disgust, fear, and surprise). Situational prompts were then generated from three sources: narratives elicited through interviews with five actors, cues from prior research, and interactions with ChatGPT 4. After revision by two psychology experimenters, 115 preliminary prompts were obtained, with approximately equal representation across emotion categories.

Next, 28 college students evaluated each prompt by indicating its primary emotion category and perceived intensity, with both single and multiple responses allowed. Preliminary analyses indicated that the primary endorsement rate for each item exceeded 85% within its intended emotion category. Items were screened and initially ordered based on the extent to which their mean intensity ratings matched the target levels, with a standard deviation close to 1. This screening process retained 55 items. Then, 35 additional scenarios were developed or revised, yielding a final pool of 90 items divided into three preliminary sets of emotional scenario texts.

Finally, the three sets were validated sequentially. In total, 86 participants ranked the scenarios within each set in ascending order of perceived emotional intensity. Scenarios were retained if mean ratings followed the intended intensity hierarchy and the modal response corresponded closely to the target level. Minor discrepancies were corrected through textual refinement, whereas substantial deviations resulted in re-evaluation and replacement. This procedure produced three validated sets of emotional scenario texts, designated T1, T2, and T3.

#### 2.1.2. Actors

Three male and three female actors were recruited for the filming of the emotional expression database. All actors were senior students enrolled in professional performing arts academies in China, aged between 21 and 22 years, and were appropriately compensated for their participation in the study.

#### 2.1.3. Shooting Process

Filming will be carried out in three phases (Q1–Q3), with one male and one female actor included in each phase. One week before filming, each actor will receive a set of scenario texts and will be instructed to familiarize themselves with the materials and prepare their performances independently.

During filming, three cameras positioned at the front center, slight right, and slight left angles will record simultaneously, accompanied by professional audio equipment. The examiner will read the scenario prompts aloud on site. Once ready, actors will signal the start of filming by raising a hand and will then produce continuous emotional expressions corresponding to the target emotion category and intensity level. Emotions will be performed in ascending order of intensity, with male and female actors alternating to facilitate preparation and emotional transitions.

Each actor will portray six basic emotions at five intensity levels. For every intensity level, two performances will be recorded: a vocal-expression performance and a non-vocal-expression performance, each with a minimum duration of 20 s. During vocal-expression performances, actors will utter random numbers lacking emotional semantic meaning. During non-vocal-expression performances, emotions will be expressed exclusively through facial expressions and body movements.

#### 2.1.4. Post Processing

To reduce participant fatigue in subsequent experiments, each video clip was edited to a duration of 10–20 s. From each original recording, actors independently selected the continuous segment that best represented the intended emotion category and intensity level. Selection was based on professional acting judgment, prioritizing segments that showed the most complete emotional expression, the clearest hierarchical progression in intensity, and intact vocal information. The procedure yielded a corpus of emotional expression clips produced by six actors and recorded from three camera angles. Following post-processing into two versions for each clip—an original-audio version and a muted version—the final Chinese Multimodal Emotion Expression Database contained 2160 video recordings.

#### 2.1.5. Validation of Stimuli

Based on the requirements of subsequent studies, frontal-view videos were selected from the database under vocal-expression (WQYS), muted vocal-expression (YSJY), and muted non-vocal-expression (WSJY) conditions. A total of 540 recordings were then subjected to expert validation and evaluation by general participants to examine whether the actors’ emotional expressions were consistent with the intended emotion categories.

#### 2.1.6. Procedure

- Expert Evaluation. The six performers involved in the filming constituted an expert panel and were asked to conduct cross-evaluations of one another’s performances. Each performer evaluated the performances of three other performers. To avoid possible interference from memory of simultaneous live performances, performers who were filmed together were not assigned to evaluate each other. Each evaluator rated video clips of three other performers expressing six basic emotions across all intensity levels under the three recognition conditions, for a total of 270 clips.
- Participant Evaluation. In total, 90 participants were randomly assigned to one of three groups (Q1–Q3) to evaluate the video clips. Each participant rated 180 clips, including 60 clips in each of the three recognition conditions (WQYS, YSJY, and WSJY), drawn from two actors within the assigned group. After data screening, 29 valid cases were retained in Q1, 29 in Q2, and 28 in Q3. The final sample therefore consisted of 86 participants. Participants were aged 18–30 years, with a mean age of 22.03 years (SD = 2.73). To mitigate potential learning and fatigue effects, evaluations under the three recognition conditions were conducted in three separate sessions, with a minimum interval of 8 h between sessions. Because the video content was identical in the WQYS and YSJY conditions, two counterbalanced presentation orders were used: (1) WQYS–WSJY–YSJY and (2) YSJY–WSJY–WQYS. Participants within each group were randomly assigned to one of the two orders.

#### 2.1.7. Rating

- Expert Evaluation. For each video clip, expert raters completed three judgment tasks related to emotional category and intensity: identifying the primary emotion category in the video (single choice), rating the intensity of that emotion (single choice, 1-mild to 5-extreme), and indicating whether any additional emotions were present (multiple responses permitted). The inclusion of the third task was intended to reduce systematic measurement error associated with forced single-choice judgments in cases of ambiguous or blended emotional expressions.
- Participant Evaluation. For each video clip, participants completed two judgment tasks concerning emotional category and intensity: identifying the primary emotion expressed in the video (single choice) and rating the intensity of the expressed emotion (single choice, 1-mild to 5-extreme).

#### 2.1.8. Analyses

- Expert Evaluation. Kendall’s coefficient of concordance (Kendall’s W) was used to assess inter-rater agreement among experts for both emotional category and emotional intensity ratings. The expert ratings were then integrated across the three evaluators: for each video clip, emotional category endorsed by at least two of the three evaluators was taken as the final category judgment, and the mean intensity rating was used as the final intensity judgment. Finally, agreement between the integrated expert judgments and the expected emotional category was assessed.
- Participant Evaluation. For each video clip, the expected recognition rate was operationalized as the proportion of participants who identified the intended emotional category:
  expected recognition rate = *n*_score1_/*n*,
  Here, *n* represents the total number of respondents, and *n*_score1_ denotes the number of respondents who correctly identified the expected emotional category. For the analysis of emotional intensity, only responses that matched the expected emotional category were retained. Based on these responses, the mean and standard deviation of the rated emotional intensity were calculated for each video clip. Subsequently, two one-way analysis of variance (ANOVA) was performed, with the preset intensity level as the independent variable and the mean and standard deviation of participant-rated intensity as the dependent variables.
- Video Scoring Rules. Expert judgments were used as the scoring key for each video clip. For emotional category, the correct answer was defined as the category endorsed by at least two of the three experts. For emotional intensity, the correct answer was defined as the mean intensity rating across the experts. Participant responses to the emotional category item were scored dichotomously: responses matching the correct category received 1 point, whereas all other responses received 0 points. Participant responses to the emotional intensity item were scored using a three-level rule. Let s represent the participant’s intensity rating and x represent the expert-defined correct intensity. If s ∈ [x − 0.5, x + 0.5], the response received 2 points; if s ∈ [x − 1.5, x − 0.5) ∪ (x + 0.5, x + 1.5], the response received 1 point; otherwise, it received 0 points.
- Video Difficulty Calculation. The difficulty of emotional category for each video clip was calculated as the proportion of correct responses among all responses. The difficulty of emotional intensity was calculated as the mean participant intensity score divided by the maximum possible score. The overall video difficulty index was defined as the average of the emotional category and emotional intensity indices.

### 2.2. Results

#### 2.2.1. Expert Evaluation

As shown in Table 1, the Kendall’s W coefficients for inter-expert agreement on emotional category ranged from 0.56 to 0.79 (all *p* < 0.001), whereas those for emotional intensity ranged from 0.59 to 0.82 (all *p* < 0.001). These results indicate good agreement among the three experts in their evaluations of the 90 video clips produced by each actor. In addition, the Kendall’s W coefficients between the integrated expert judgments and the preset video outcomes ranged from 0.78 to 0.90 for emotional category (all *p* < 0.001) and from 0.81 to 0.92 for emotional intensity (all *p* < 0.001), suggesting that the filmed emotional expression database largely satisfied the preset design requirements. Overall, 94.81% of the videos showed consistency between the integrated emotional category and the preset emotional category.

#### 2.2.2. Participant Evaluation

**Figure 1:**
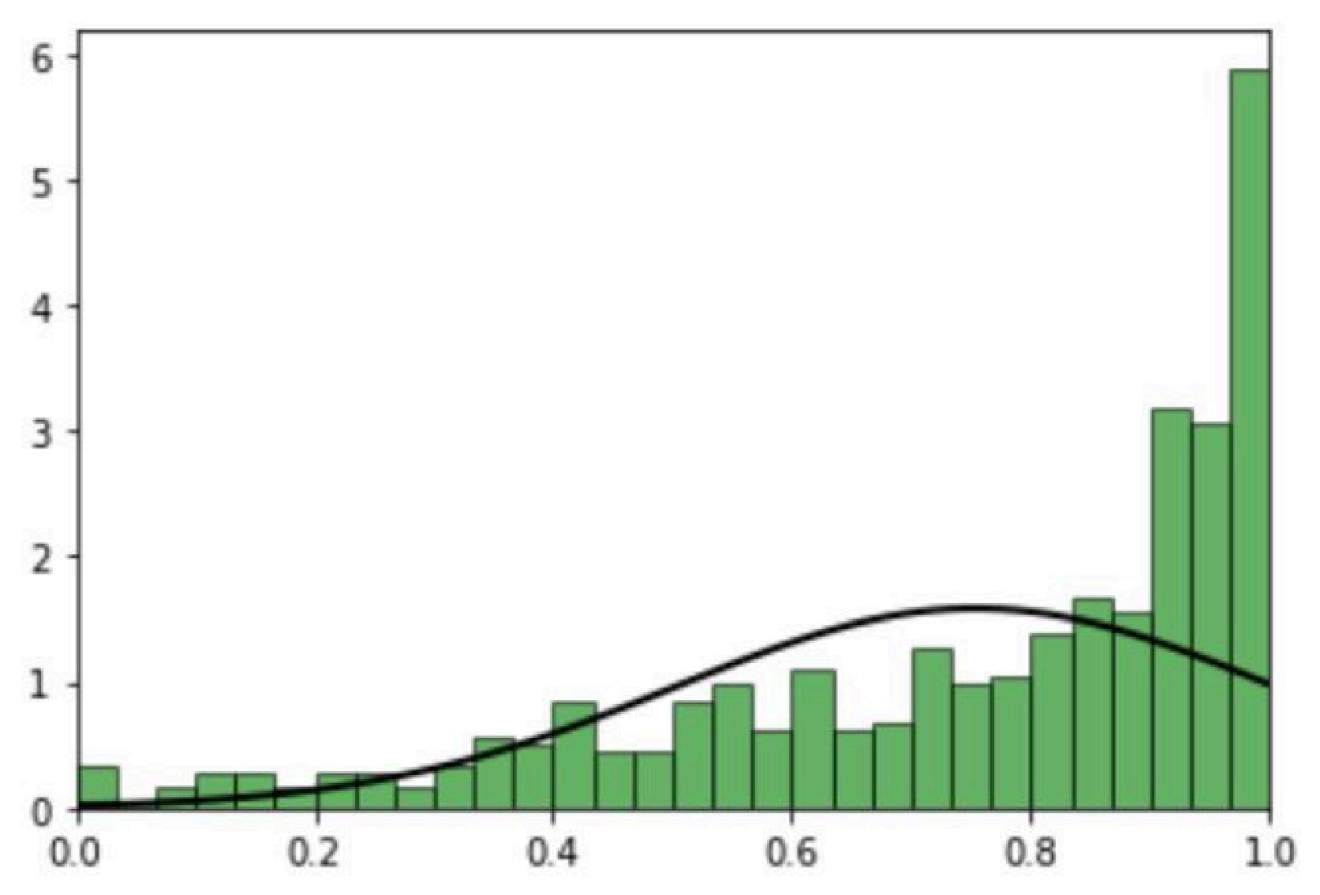
Histogram of emotion category recognition rates.

As shown in Figure 1, most of the 540 videos achieved satisfactory recognition rates for the target emotion categories, with a mean of 0.75 (SD = 0.25). Recognition rates ranged from 0 to 1, with quartiles of 0.59, 0.84, and 0.97. These results indicate that the filmed emotion expression database largely met the preset requirements.

**Figure 2:**
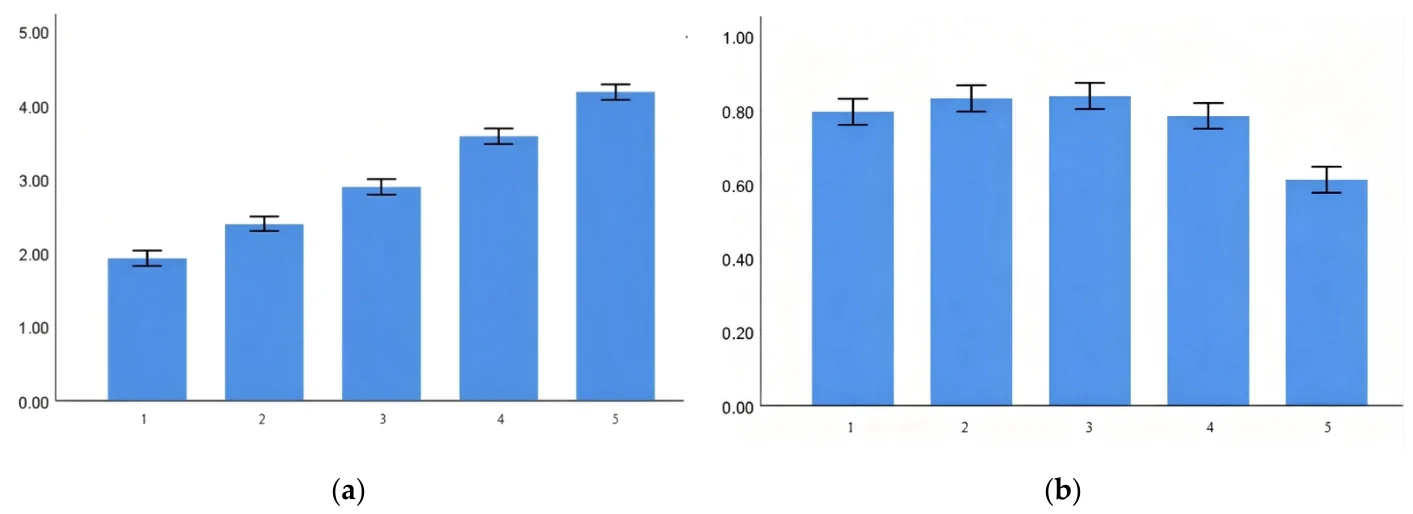
(**a**) Mean values for each of the five intensity levels; (**b**) standard deviations for each of the five intensity levels.

The mean emotional intensity ratings generally corresponded to the preset intensity levels, with distinct mean values observed across levels and a progressive increase as the preset level increased. A one-way ANOVA on mean emotional intensity revealed a significant main effect of performance level, F (4, 529) = 291.33, *p* < 0.001. Post hoc comparisons showed significant differences in intensity ratings across all levels (M1 = 1.94, M2 = 2.42, M3 = 2.93, M4 = 3.61, M5 = 4.17; all *p*s < 0.001), as shown in Figure 2a. These results indicate that actors’ performances exhibited clear intensity differences under the guidance of the preset performance tiers.

A one-way ANOVA on the standard deviation of emotional intensity across videos also revealed a significant main effect of performance level, F (4, 528) = 27.03, *p* < 0.001. Post hoc comparisons showed that the standard deviation at the “5-Extreme” intensity level was significantly lower than that at the other four levels (M1 = 0.80, M2 = 0.82, M3 = 0.83, M4 = 0.78, M5 = 0.61; all *p*s < 0.001), as shown in Figure 2b. This suggests that as emotional intensity increased, ratings of the identified emotion became more concentrated.

#### 2.2.3. Video Difficulty Distribution

**Figure 3:**
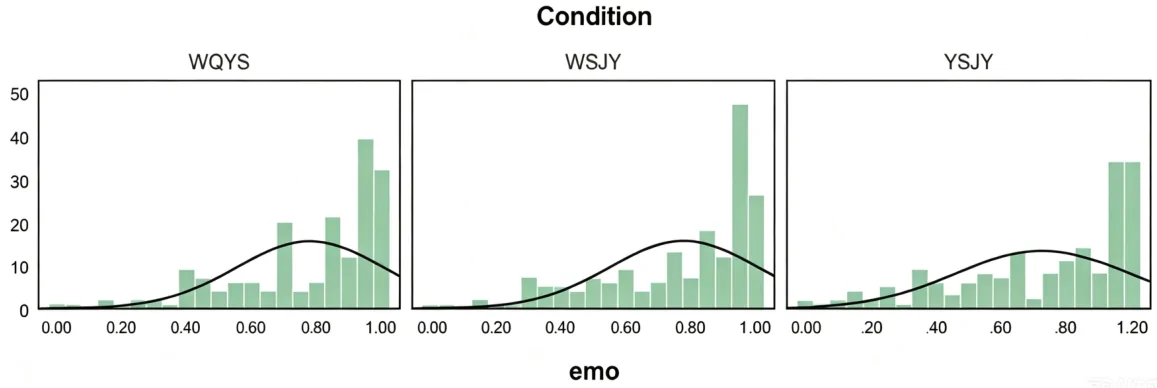
Histogram of emotion category difficulty under three recognition conditions. WQYS means vocal-expression, WSYS means muted non-vocal-expression, and YSJY means muted vocal-expression.

As shown in Figure 3, the distribution of emotion category difficulty across the three recognition conditions ranged from 0 to 1 and was generally negatively skewed, and recognition of emotion categories in the overall video emotion dataset was relatively easy.

**Figure 4:**
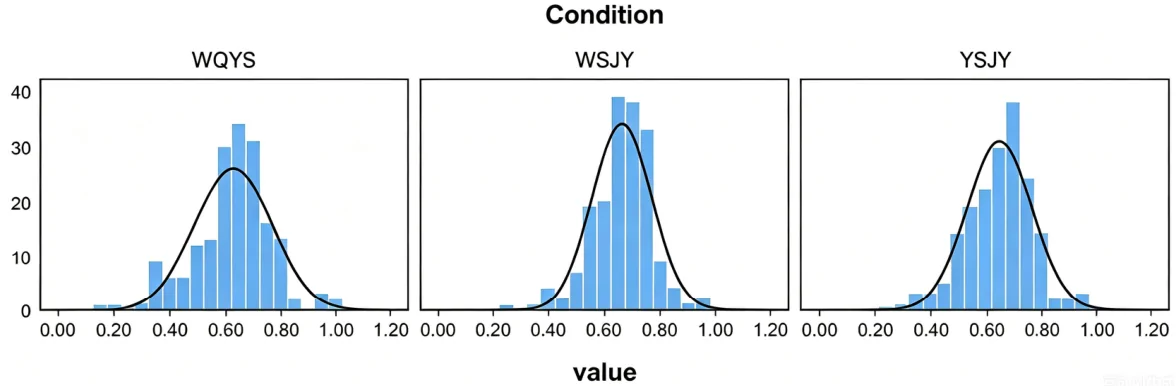
Histogram of emotion intensity difficulty under three recognition conditions. WQYS means vocal-expression, WSYS means muted non-vocal-expression, and YSJY means muted vocal-expression.

As shown in Figure 4, the distribution of emotional intensity difficulty across the three recognition methods ranged from 0 to 1 and approximated a normal distribution. This suggests that the difficulty of judging emotional intensity was relatively balanced across the video materials, spanning levels from relatively easy to relatively difficulty.

### 2.3. Discussion

In Study 1, we constructed and validated a localized dynamic multimodal emotion expression database featuring Chinese actors as expressers. The results demonstrated good internal validity of the materials, as reflected in the strong consistency among expert judges in both emotion category and intensity recognition, as well as the high agreement between the integrated expert judgments and the intended targets. Subsequently, using the experts’ recognition judgments as the scoring criterion, we evaluated the validity of the material database with a general participant sample. The results showed that participants achieved relatively high recognition rates for emotion categories. Previous research has found that actors outperform non-actors in facial expression recognition (Conson et al., 2013), suggesting that using actors’ recognition responses as the criterion is reliable. In addition, participants’ intensity ratings generally corresponded to the intended five-level intensity hierarchy, with significant differences observed between adjacent levels. This further indicates that the actors were able to consistently portray emotional expressions at the required intensity levels.

We also found that the dispersion of ratings at the highest intensity level was significantly lower than that at the other levels, suggesting that participants’ judgments of emotional intensity became more consistent as intensity increased. This pattern may be related to the fact that at extreme intensity levels, emotional cues become highly salient and unambiguous, whereas at lower intensity levels, the cues are more subtle and thus more difficult to recognize. Previous research has demonstrated that facial expression recognition depends on intensity (Favelle et al., 2026). Our finding that participants showed differential recognition performance across intensity levels provides indirect support for the importance of incorporating intensity recognition into the assessment of individual differences in emotion recognition ability. Overall, Study 1 demonstrates that the video database we constructed not only reliably conveys information about emotion category and intensity, but also exhibits a hierarchical difficulty structure, thereby providing a foundation for the test development undertaken in Study 2.

## 3. Study 2

In Study 2, we aimed to develop and validate a multimodal emotion recognition ability test based on the multimodal emotion expression video database established in Study 1. To ensure that the test items could effectively differentiate individuals across different ability levels, we selected initial test items from the three recognition conditions based on item difficulty, and participants were required to judge both the emotion category and the emotional intensity expressed in each video. Subsequently, we further verified the item difficulty and refined the item set based on reliability and discrimination indices. Next, we assessed the preliminary criterion-related validity of the test by examining its associations with theoretically relevant external variables, including emotion understanding, depression, alexithymia, and autistic traits. Finally, we investigated the construct validity of the test by comparing three alternative factor structures to determine which best represented the underlying structure of the test.

### 3.1. Methods

#### 3.1.1. Participants

In total, 241 participants were recruited for the online experiment. After screening for response time, accuracy, and performance on verification and lie-detection items, 236 valid responses were retained for analysis. Of these participants, 123 were male and 113 were female, with ages ranging from 18 to 30 years (M = 21.94, SD = 2.76).

#### 3.1.2. Measures

Multimodal Emotion Recognition Ability Test. The test was developed using 90 video stimuli selected from Study 1, with 30 videos drawn from each of three recognition conditions. The distribution of videos across the six target emotion categories was relatively balanced, and the overall item difficulty (see Table S1) distribution did not significantly deviate from normality (K^2^ = 3.25, *p* = .20). Further details regarding stimulus selection and video characteristics are reported in Table S2. Each video required participants to complete two tasks: identifying the target emotion category and judging the level of emotional intensity. To capture emotion recognition ability more comprehensively, scores from these two tasks were integrated into a single difficulty-weighted index. The score for each video was calculated as follows: Each video required participants to complete two tasks: identifying the target emotion category and judging the level of emotional intensity. To capture emotion recognition ability more comprehensively, scores from these two tasks were integrated into a single difficulty-weighted index. The rationale for this weighting approach is grounded in item response theory (IRT): because the video materials vary considerably in difficulty, a simple sum of correct answers would disproportionately reflect performance on easier items rather than the individual’s underlying ability. According to IRT principles, more difficult items carry greater information about individual differences in ability and should therefore be assigned larger weights in the total score. Furthermore, each video comprises two distinct dimensions—emotion category recognition and emotion intensity recognition—which have different response scales. To prevent the intensity component from dominating the composite score, we calibrated the intensity score so that the two dimensions contribute equally to the total. Accordingly, the recognition score for each video was calculated as follows:

(1) $${\mathrm{Score}}_{\mathrm{video}}=\sqrt{1-p1}\times {\mathrm{Score}}_{1}+(\sqrt{1-p2}\times {\mathrm{Score}}_{2})/2$$

Score_1_ and Score_2_ denote the participant’s scores on the emotion category and the emotional intensity, respectively, for a given video, whereas *p*1 and *p*2 represent the corresponding task difficulty values. The resulting Score_video_ ranged from 0 to 2. A participant’s overall test score was defined as the mean of Score_video_ across all 90 videos.

To evaluate criterion-related validity, the normative measures included in the present study assessed emotion understanding, social skills, and mental health problems.

- Situational Test of Emotional Understanding–Brief, STEU-B (Allen et al., 2014). STEU-B is a 19-item performance-based measure of emotional understanding in which participants read brief emotion-related scenarios and select the most appropriate response from five options. Responses are scored dichotomously (1 = correct, 0 = incorrect). In the present sample (n = 236), the original 19-item version showed relatively low internal consistency (Cronbach’s α = 0.493). Accordingly, reliability testing and IRT analyses were conducted to optimize the scale. After removing Items 5, 4, 13, 19, and 10, the remaining 14 items demonstrated acceptable fit to a 2PL IRT model, χ^2^ (77) = 90.09, *p* = .15, RMSEA = 0.027, CFI = 0.92, TLI = 0.91, SRMR = 0.060. Discrimination parameters ranged from 0.163 to 2.339, and difficulty parameters ranged from −2.829 to 5.948. All retained items showed adequate fit (all *p* > 0.05). The modified version produced a Cronbach’s α of 0.514. STEU-B scores were operationalized as the participant ability estimates obtained from the 2PL model.
- Interaction Anxiety Scale, IAS (Leary & Kowalski, 1993). The revised IAS includes 13 items, with 3 items reverse scored. Participants respond on a 5-point Likert scale from 1 to 5. Item scores are averaged after reverse coding, with higher scores reflecting greater social anxiety. Internal consistency in the present sample was excellent (Cronbach’s α = 0.945).
- Beck Depression Inventory–II, BDI-II (Upton, 2013). BDI-II includes 21 items, each rated on a 4-point scale from 0 to 3. Scores are summed across items, with higher scores reflecting more severe depressive symptoms. Internal consistency in the present sample was excellent (Cronbach’s α = 0.908).
- Abridged Version of the Autism-Spectrum Quotient, AQ-Short (Hoekstra et al., 2011). AQ-Short includes 28 descriptive statements assessing personal preferences and habitual tendencies. Participants respond on a 4-point Likert scale from 1 to 4, with 15 items reverse scored. Item scores are summed, with higher scores reflecting higher levels of autistic traits. Internal consistency in the present sample was acceptable (Cronbach’s α = 0.766).
- Toronto Alexithymia Scale, TAS-20 (Taylor et al., 1992). TAS-20 includes 20 items rated on a 5-point Likert scale from 1 to 5. Total scores range from 20 to 100, with higher scores indicating greater alexithymia. Internal consistency in the present sample was good (Cronbach’s α = 0.843).

#### 3.1.3. Procedure

All assessments were administered online via a questionnaire platform. Prior to the formal assessment, participants were instructed to read the detailed instructions carefully and ensure that they fully understood the task requirements before proceeding. Participants first completed the multimodal emotion recognition ability test, which required approximately 45 min. After completing this test, they were allowed to take a short break before proceeding to the remaining questionnaires, which took approximately 15 min to complete.

### 3.2. Results

#### 3.2.1. Video Descriptive Statistics

- Item Difficulty Validation. The distribution, range, mean, standard deviation, and results of normality tests for the difficulty of the video materials under the three recognition conditions are reported in Figure S1. The difficulty scores were approximately normally distributed across all conditions, with mean values ranging from 0.48 to 0.50. A one-way ANOVA further indicated that there were no significant differences in overall video material difficulty across the three recognition conditions, F (2, 87) = 0.09, *p* = .91. Likewise, no significant differences were found in category recognition difficulty, F (2, 87) = 0.20, *p* = .82, or in intensity-recognition difficulty, F (2, 87) = 0.82, *p* = .42. These results indicate that the test materials met the prespecified screening criteria.
- Material Reliability. Table 2 presents the Cronbach’s α coefficients for the overall test and its subcomponents. The overall test showed good internal consistency (α = 0.88). The emotion category recognition dimension yielded an α of 0.61, whereas the emotion intensity recognition dimension showed excellent internal consistency (α = 0.91). Across recognition conditions, the reliability coefficients for the emotion intensity recognition dimension ranged from 0.74 to 0.83. By contrast, the reliability coefficients for the emotion category recognition dimension ranged from 0.34 to 0.41. These results suggest that the test may require further refinement, particularly with respect to the emotion category recognition dimension.

**Figure 5:**
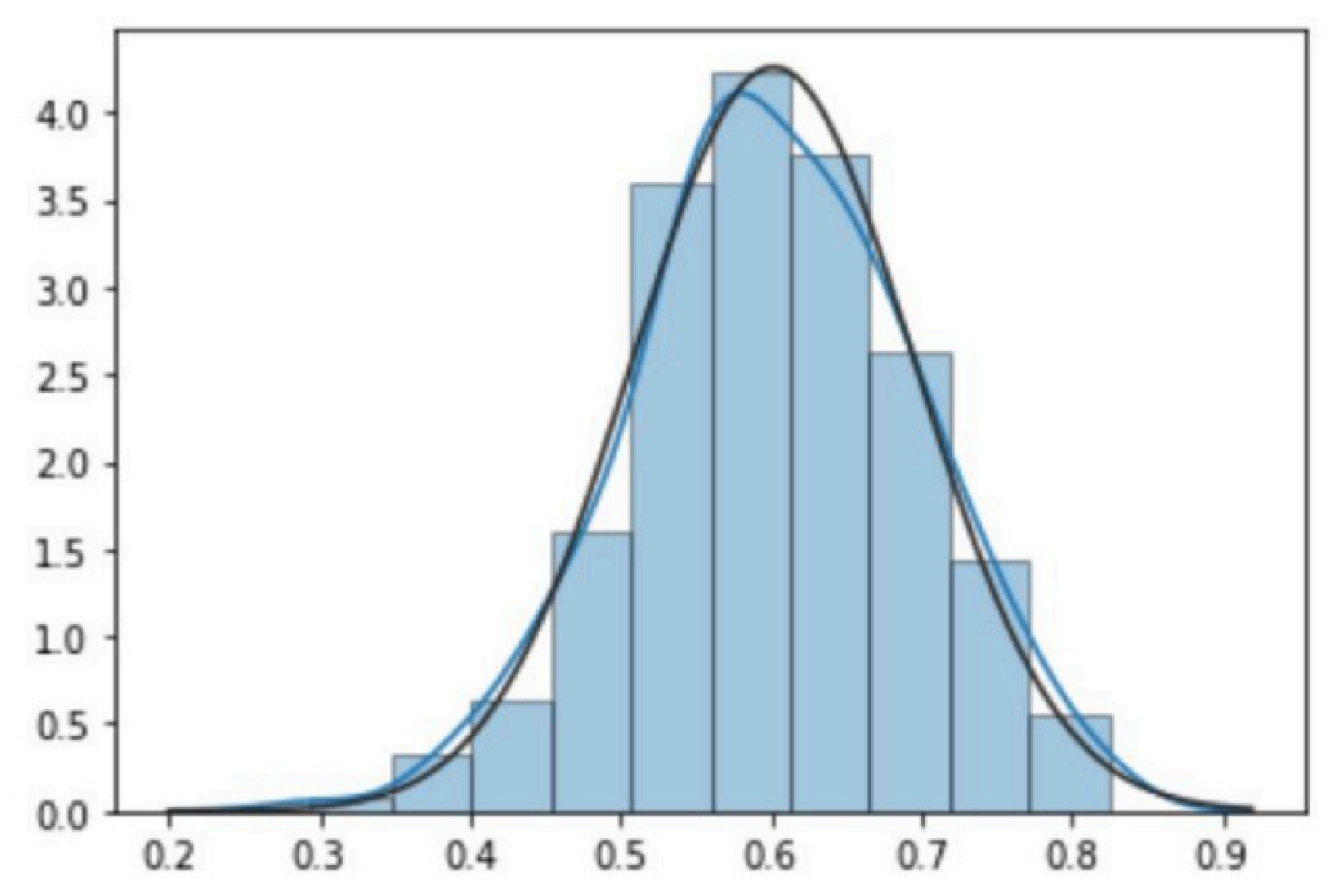
The distribution of subjects’ total test scores.

Distribution of Test Subject Scores. Participants’ scores on both emotion category recognition and emotion intensity recognition were approximately normally distributed (see Figure S2). The correlation between these two variables was not significant, r = 0.077, *p* = 0.239, and their association remained weak across different recognition conditions (see Table S3). These findings suggest that the two types of recognition may reflect relatively independent aspects of ability, although both are theoretically regarded as essential components of emotion recognition ability. After weighting, the distribution of participants’ total test scores is shown in Figure 5. The total scores were approximately normally distributed, with M = 0.602, SD = 0.094, K^2^ = 0.677, *p* = 0.713, and ranged from 0.295 to 0.825. These results indicate that the total test score demonstrated good discriminative ability. Subsequent analyses of test quality were therefore conducted based on the integrated total score.

#### 3.2.2. Test Reliability and Discrimination

After weighted scoring, the Cronbach’s α coefficients are presented in Table 3. The initial version of the test showed an overall reliability of 0.82, with reliability coefficients ranging from 0.58 to 0.69 across different recognition conditions. The reliabilities of the individual subtests indicated room for improvement. Accordingly, each subtest was revised primarily to improve Cronbach’s α coefficients while also taking item quantity and item discrimination into account. Following revision, 61 videos were retained (see Table S4). The modified test achieved an overall reliability of 0.86, with reliability coefficients ranging from 0.70 to 0.73 across different recognition conditions, indicating improved internal consistency.

**Figure 6:**
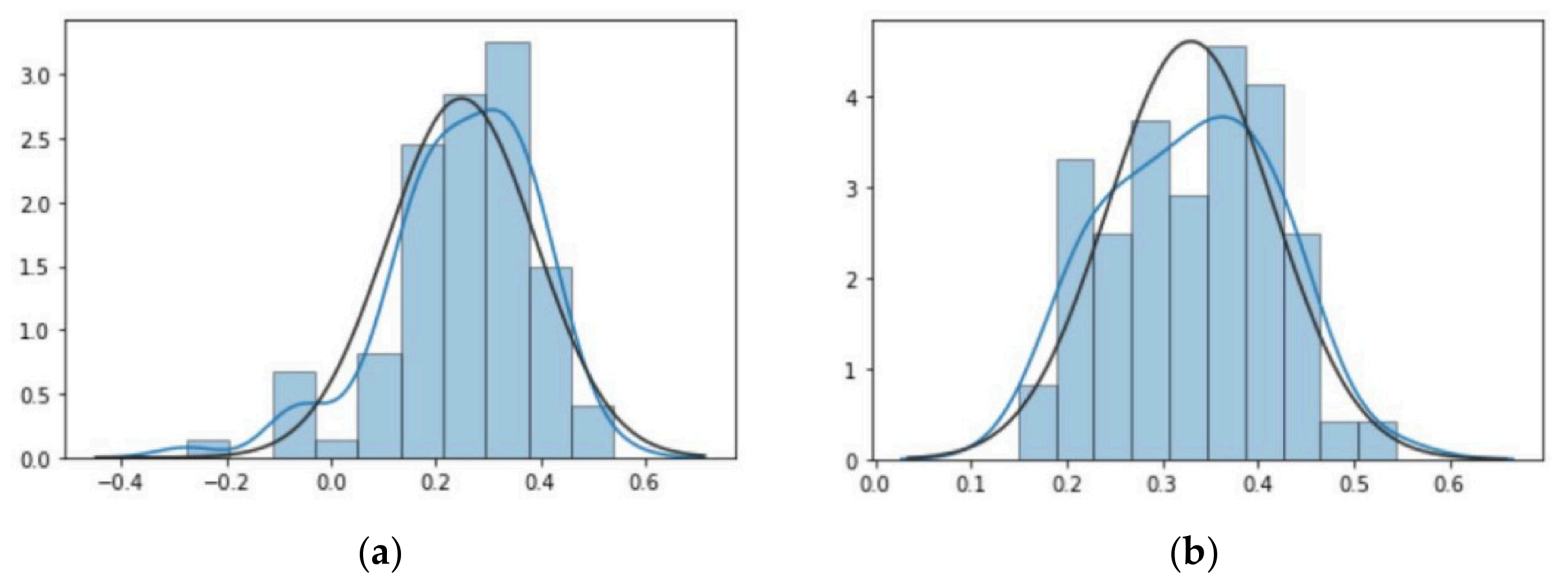
(**a**) Distribution of item discrimination for the preliminary test; (**b**) distribution of item discrimination for the optimized test.

Item discrimination in the preliminary test ranged from −0.28 to 0.54 (M = 0.25, SD = 0.14), and its distribution deviated significantly from normality, K^2^ = 15.69, *p* < .001, as shown in Figure 6a. In addition, several items showed negative discrimination values. In the optimized test, item discrimination ranged from 0.15 to 0.54 (M = 0.33, SD = 0.09), and the distribution was approximately normal, K^2^ = 1.86, *p* = .40, as shown in Figure 6b. These results indicate that the modified test demonstrated substantially improved item discrimination.

#### 3.2.3. Criterion-Related Validity

Table 4 shows the correlations between subjects’ total test scores on the initial test and the criterion measures. Total test scores were positively associated with emotion understanding ability (r = 0.162) and negatively associated with depression (r = −0.170), indicating that higher emotion recognition ability was related to better emotion understanding and lower depression. Across recognition conditions, different patterns of correlation emerged. Muted vocal-expression (YSJY) scores were positively correlated with emotion understanding (r = 0.191) and negatively correlated with alexithymia (r = −0.154) and depression (r = −0.192). Muted non-vocal-expression (WSJY) scores were negatively correlated with depression (r = −0.173). No significant correlations were found between vocal-expression (WQYS) scores and any of the school-based measures.

Table 5 shows the correlations between subjects’ total test scores on the optimized test with the criterion measures. Total test scores were negatively associated with depression (r = −0.174) and autistic traits (r = −0.134), suggesting that greater emotion recognition ability was associated with lower depression and fewer autistic traits. Across recognition conditions, a similar pattern was observed. Muted vocal-expression (YSJY) and muted non-vocal-expression (WSJY) conditions were negatively correlated with alexithymia, depression, and autistic traits, whereas scores in the vocal-expression (WQYS) condition were not significantly correlated with any of the criterion measures.

#### 3.2.4. Construct Validity

**Figure 7:**
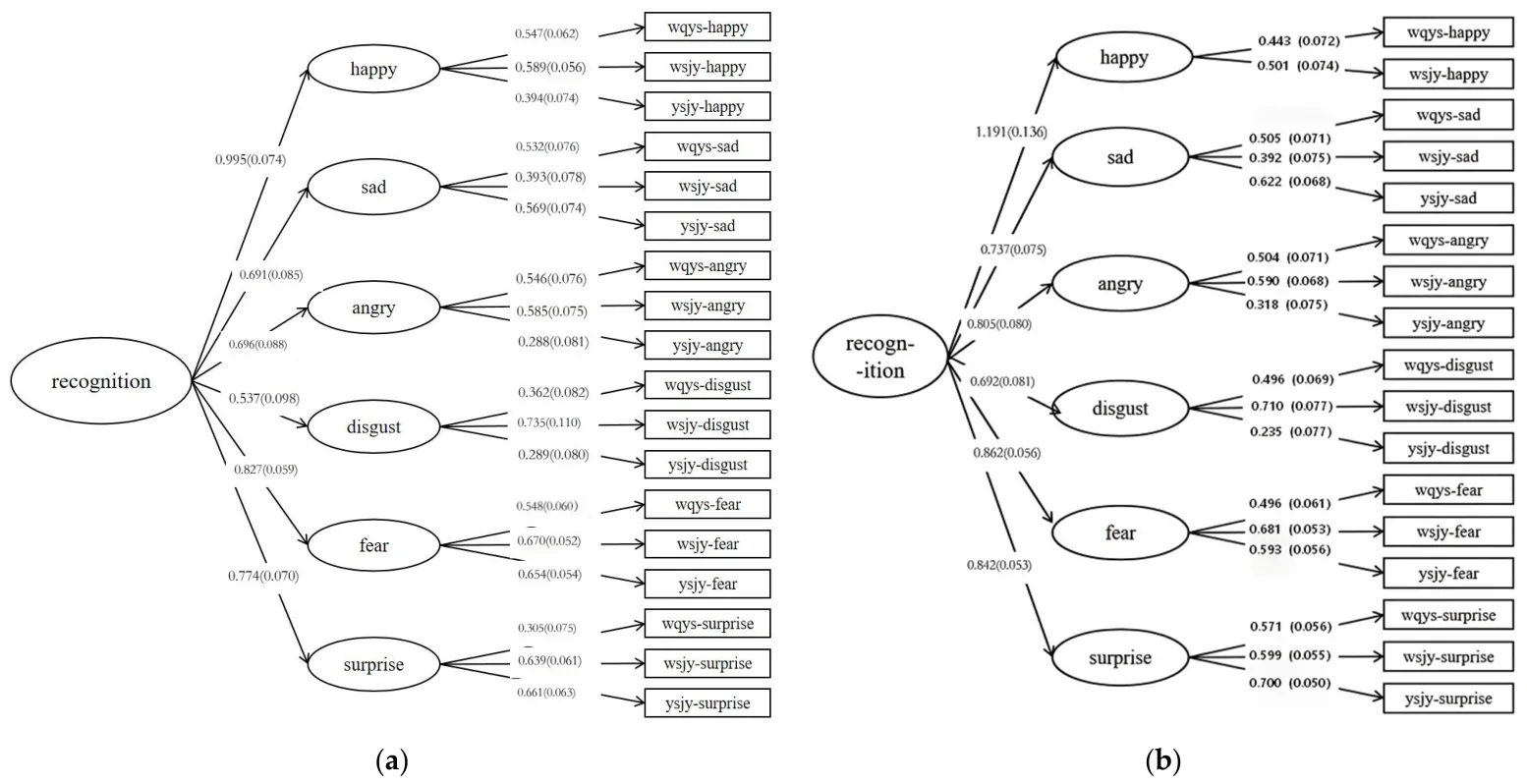
Second-order models for the preliminary and optimized tests: (**a**) second-order models for the preliminary tests; (**b**) second-order models for the optimized tests.

Three alternative models were tested for the preliminary test: a single-factor model, a second-order model structured by recognition conditions, and a second-order model structured by emotion categories. Fit indices for these models are reported in Table 6. Model 3, the second-order emotion-category model, demonstrated the best overall fit. Standardized factor loadings for this model are shown in Figure 7a.

Similarly, three alternative models were tested for the optimized test: a single-factor model, a second-order model structured by emotion categories, and a second-order model structured by emotion recognition. The corresponding fit indices are reported in Table 7. Among these, model 3, the second-order emotion-recognition model, showed the best fit. Standardized factor loadings are displayed in Figure 7b. Taken together, these findings suggest that different emotion categories may function as distinct first-order factors that are organized under broader higher-order emotion recognition factors.

### 3.3. Discussion

In Study 2, we developed and refined a multimodal emotion recognition test based on the stimulus database established in Study 1 and introduced a scoring method that integrated emotion category and intensity. Results showed that the difficulty distributions of the 90 videos across the three recognition conditions were approximately normal, with no significant differences among conditions in overall difficulty, category recognition difficulty, or intensity recognition difficulty, indicating that the selected materials were well comparable across conditions.

Next, we conducted reliability analyses. The initial test demonstrated acceptable overall reliability; however, the reliability of the emotion category recognition subscale remained relatively low even after item refinement. This may be attributed to the fact that category judgments are more susceptible to individual interpretive variability (Mortillaro & Schlegel, 2023; Russell, 1980). In addition, the modest reliability of category recognition may reflect the inherent limitations of mapping dynamic, multidimensional emotional displays onto discrete categorical labels (Bänziger et al., 2012; Chen et al., 2024).

Regarding validity, the optimized test showed no significant correlation with emotion understanding ability. This may be due to the fact that during test optimization, items with poor discrimination were removed. Although this improved internal consistency to some extent, it also narrowed the content breadth of the test, thereby attenuating its correlation with emotion understanding. Nevertheless, significant negative correlations were found with depression and autistic traits, providing preliminary evidence for criterion-related validity. This pattern is broadly consistent with previous findings (Connolly et al., 2020; Laukka et al., 2021). With respect to depression, prior research has documented impairments in basic emotion recognition among depressed individuals, with particularly pronounced difficulties in recognizing low-arousal facial expressions (Csukly et al., 2009). Depressed individuals have also been found to misattribute low- to moderate-arousal emotions as high-arousal emotions, suggesting that biases in emotional information processing may emerge across both early and later stages of cognition. Such biases may, in turn, contribute to enhanced sensitivity to sadness-related stimuli (Li et al., 2023). Regarding autistic traits, our findings are also in line with evidence that individuals with autism spectrum disorder tend to show lower emotion recognition accuracy (Masoomi et al., 2024).

Finally, construct validity analyses showed that the second-order emotion category model provided the best fit, suggesting that the test primarily captures variance attributable to differences in processing specific emotions rather than differences in processing information channels. This finding is consistent with the view that emotion recognition ability is a supramodal factor—that is, individuals who are skilled at recognizing emotions from facial cues also excel at identifying emotions from non-facial cues, such as those conveyed through the body (Lewis et al., 2016). Overall, Study 2 demonstrates that the test possesses acceptable reliability, item discrimination, preliminary criterion-related validity, and construct validity, and can effectively capture emotion recognition performance across different information conditions.

## 4. General Discussion

In summary, the present study constructed a localized multimodal emotional expression database suited to the Chinese cultural context and developed a corresponding assessment tool for multimodal emotion recognition. The results provide converging evidence that the tool is psychometrically sound and capable of assessing both emotion category recognition and emotion intensity recognition under different recognition conditions. By incorporating multimodal emotional information and more naturalistic recognition contexts, this work extends existing approaches to emotion recognition assessment and improves ecological validity. Overall, the findings suggest that the proposed tool offers a useful and context-sensitive method for evaluating individuals’ emotion recognition ability, with potential value for future research and applied assessment.

### 4.1. Limitations and Future Prospects

Despite the progress made in database construction and test development, the present study still has several limitations. Most notably, the multimodal database was constructed on the basis of basic emotion theory and included only six basic emotions. Although emotional intensity was further differentiated using a five-level scale, dynamic neutral expressions and complex emotions were not systematically examined. Neutral stimuli may be particularly sensitive to recognition bias (Surguladze et al., 2004) and complex emotions contain rich social information beyond what can be captured by the basic emotion framework (Keltner & Buswell, 1997). At the same time, the inclusion of complex emotions remains challenging because of inconsistencies in their theoretical definition and the subjective nature of their identification. Future studies should therefore seek to expand the database by progressively incorporating neutral and complex emotions within a more clearly defined theoretical framework, thereby improving both its comprehensiveness and its applied value.

Second, the validation of the video materials and the assignment of correct labels in this study relied on actors’ own identification responses. Although actors may possess stronger emotion recognition ability than non-experts due to their professional training and experience (Conson et al., 2013; Goldstein et al., 2009), their judgments are still likely to reflect a degree of subjective bias. Moreover, in the present study, actors cross-evaluated each other’s performances, the emotion generation processes of different actors share certain similarities. Consequently, the correct answers derived from actors’ identifications may, to some extent, be influenced by these similar generation processes. In view of this, future research could explore alternative scoring criteria—for example, using the mode or mean of participant responses as the standard answer, depending on the purpose of the assessment, or recruiting independent experts to provide ratings that serve as the final scoring benchmark. Alternatively, a combined approach that integrates expert evaluations with aggregated responses from a large sample may be particularly useful for establishing scoring standards that are both stable and sensitive to contextual factors.

When examining the psychometric properties of the video database in Study 1, only 90 participants were recruited and assigned to separate groups for evaluation, with each video material being assessed by approximately 30 participants. This relatively small sample size per video may not provide sufficiently stable estimates for the measurement properties of the materials. Future applications and validation studies of the database could consider increasing the sample size to enhance the stability and generalizability of the labeling results.

Additionally, although this study proposed an integrated scoring method that combines emotion category recognition and intensity recognition, the relative weighting of these two components was based primarily on item difficulty and therefore still requires stronger theoretical and statistical support (Mortillaro & Schlegel, 2023). Notably, the correlation between emotion category scores and emotion intensity scores was not significant (r = 0.077). Apart from differences in scoring methods, this result may also imply that the two types of tasks rely on different psychological processes. Nevertheless, under such circumstances, combining two low-correlated dimensions into a single total score may carry the risk of information loss, which could obscure the unique contributions of each dimension. Therefore, future research should integrate response time, eye-tracking, or neural indicators to further examine the similarities and differences in cognitive processing mechanisms between the two tasks. In addition, future studies with larger samples could employ bifactor models or multidimensional item response theory to test whether a common general emotion recognition factor can account for the two dimensions, and to optimize the scoring method by more precisely modeling the weighting and contribution mechanisms of different item types.

### 4.2. Implications

The present study makes an important contribution by developing a new localized multimodal tool for assessing emotion recognition ability in the Chinese cultural context. Unlike conventional tests based on static stimuli or single-category judgments, the current dynamic assessment incorporates both emotion category and intensity recognition and evaluates performance under three conditions with varying levels of information availability, thereby increasing ecological relevance. Importantly, the findings indicate that emotion recognition should not be conceptualized solely in terms of category identification. Emotion intensity recognition may represent an additional and meaningful component of this ability, highlighting the need for a more multidimensional approach to the assessment of emotion recognition.

## 5. Conclusions

The present study established a localized multimodal emotion expression database and, on this basis, developed a video-based multimodal emotion recognition ability test. Study 1 established an emotion expression database that systematically manipulates five levels of emotional intensity and three information conditions. Unlike most existing databases that rely on Western expressers and offer limited intensity gradation, the present database provides culturally appropriate, dynamically varying stimulus materials that allow researchers to examine both the categorical and intensity aspects of emotional expression simultaneously. Study 2 developed a test with sound psychometric properties based on the materials from Study 1. In addition, the second-order emotion category structure model provided empirical support for the supramodal nature of emotion recognition ability. In summary, the present study provides, methodologically, a culturally appropriate tool for assessing multimodal emotion recognition ability in Chinese populations. Theoretically, it advances the understanding that emotion recognition is not a unitary ability but a multidimensional construct. Together, these contributions offer both a practical resource for future research and a conceptual framework for rethinking how emotion recognition ability should be measured and understood.

## Figures and Tables

**Table 1:** Kendall’s W coefficients for expert evaluation and agreement with target video.

Video	Q1-F	Q1-M	Q2-F	Q2-M	Q3-F	Q3-M
Emotion Category	0.56	0.7	0.73	0.79	0.79	0.77
Emotion Intensity	0.74	0.66	0.61	0.7	0.59	0.82
Target Category	0.78	0.84	0.87	0.9	0.9	0.85
Target Intensity	0.88	0.86	0.88	0.92	0.81	0.91

Notes: The table presents Kendall’s W coefficients for the video clips of each female and male actor in each shooting group. Emotion Category indicates the agreement among experts on emotion category ratings; Emotion Intensity indicates the agreement among experts on emotion intensity ratings; Target Category indicates the agreement between the experts’ integrated category judgments and the target categories; and Target Intensity indicates the agreement between the experts’ integrated intensity ratings and the target intensities.

**Table 2:** Reliability of the category and intensity tests for the full video set and under each recognition condition.

Condition	WQYS	WSJY	YSJY	All
Category	0.41	0.34	0.4	0.61
Intensity	0.77	0.83	0.74	0.91
ALL	0.69	0.79	0.7	0.88

**Table 3:** Reliability of weighted scoring for the full video set and under each recognition condition.

	Condition	WQYS	WSJY	YSJY	All
Preliminary test	n	30	30	30	90
	Cronbach’s α	0.58	0.69	0.61	0.82
Optimized test	n	22	22	17	61
	Cronbach’s α	0.70	0.73	0.70	0.86

**Table 4:** Correlations between total scores on the preliminary test and scores under different recognition conditions with criterion measure.

Preliminary Test	M (SD)	WQYS	WSJY	YSJY	All
WQYS	0.587 (±0.105)	1.00 ***			
WSJY	0.610 (±0.123)	0.511 ***	1.00 ***		
YSJY	0.607 (±0.109)	0.546 ***	0.583 ***	1.00 ***	
All	0.602 (±0.094)	0.807 ***	0.852 ***	0.845 ***	1.00 ***
STEU-B	0.000 (±0.755)	0.123	0.095	0.191 **	0.162 *
SA	42.017 (±12.124)	0.069	−0.053	−0.029	−0.009
TT	52.844 (±10.754)	−0.021	−0.124	−0.154 *	−0.121
DP	11.823 (±10.497)	−0.053	−0.173 **	−0.192 **	−0.170 *
AQ-short	67.637 (±9.903)	−0.012	−0.122	−0.108	−0.099

Notes: * *p* < 0.05, ** *p* < 0.01, *** *p* < 0.001. WQYS denotes vocal-expression recognition condition; WSJY denotes muted non-vocal-expression recognition condition; YSJY denotes muted vocal-expression recognition condition; All denotes the total test score, STEU-B denotes emotional understanding ability, SA denotes anxiety level, TT denotes alexithymia level, DP denotes depression level, and AQ-short denotes autism traits level.

**Table 5:** Correlations between total scores on the optimized test and scores under different recognition conditions with criterion measure.

Optimized Test	M(SD)	WQYS	WSJY	YSJY	All
WQYS	0.575 (±0.137)	1.00 ***			
WSJY	0.617 (±0.154)	0.539 ***	1.00 ***		
YSJY	0.613 (±0.156)	0.505 ***	0.548 ***	1.00 ***	
All	0.601 (±0.123)	0.822 ***	0.860 ***	0.802 ***	1.00 ***
STEU-B	0.000 (±0.755)	0.107	0.066	0.128	0.118
SA	42.017 (±12.124)	0.050	−0.074	−0.069	−0.038
TT	52.844 (±10.754)	−0.005	−0.150 *	−0.143 *	−0.120
DP	11.823 (±10.497)	−0.063	−0.191 **	−0.179 **	−0.174 **
AQ-short	67.637 (±9.903)	−0.034	−0.156 *	−0.142 *	−0.134 *

Notes: * *p* < 0.05, ** *p* < 0.01, *** *p* < 0.001. WQYS denotes vocal-expression recognition condition; WSJY denotes muted non-vocal-expression recognition condition; YSJY denotes muted vocal-expression recognition condition; All denotes the total test score, STEU-B denotes emotional understanding ability, SA denotes anxiety level, TT denotes alexithymia level, DP denotes depression level, and AQ-short denotes autism traits level.

**Table 6:** CFA fit results for the preliminary test.

Model	χ^2^ (df)	CFI	TLI	RMSEA	SRMR
Model 1	231.620 (135)	0.831	0.808	0.055	0.063
Model 2	229.416 (132)	0.829	0.802	0.056	0.06
Model 3	163.088 (129)	0.940	0.927	0.033	0.051

Notes: model 1 is a single-factor model; model 2 is a second-order model structured by recognition conditions; and model 3 is a second-order model structured by emotion categories.

**Table 7:** CFA fit results for the optimized test.

Model	χ^2^ (df)	CFI	TLI	RMSEA	SRMR
Model 1	188.795 (119)	0.891	0.875	0.050	0.054
Model 2	175.420 (116)	0.907	0.891	0.047	0.052
Model 3	135.624 (113)	0.965	0.957	0.029	0.046

Notes: model 1 is a single-factor model; model 2 is a second-order model structured by recognition conditions; and model 3 is a second-order model structured by emotion categories.

## Data Availability

The datasets generated and analyzed during the current study are available from the corresponding author on reasonable request.

## Supplementary Materials

The following supporting information can be downloaded at: https://www.mdpi.com/article/10.3390/jintelligence14080169/s1, Table S1: Preliminary Difficulty of the Test Videos; Table S2: Properties of the initial test videos; Figure S1: Distribution, range, mean, standard deviation, and normality test of item difficulty; Figure S2: Distribution of Participants’ Emotion Category Scores and Emotion Intensity Scores; Table S3: Correlation between Emotion Category Scores and Emotion Intensity Scores; Table S4: Difficulty of the Optimized Test Videos.
