# Supplementary material for: Development and Validation of a Multimodal Emotion Recognition Ability Test Based on the Chinese Cultural Context
Source: J Intell. 2026 Aug 1;14(8):169. doi: 10.3390/jintelligence14080169 (PMC13514528; doi:10.3390/jintelligence14080169)
Supplement: Supplementary file 1 [file jintelligence-14-00169-s001.zip › jintelligence-4232471-supplementary.pdf]

Supplementary Materials

Table S1. Preliminary Difficulty of the Test Videos

WQYS			WSJY			YSJY		
ID	Video	Difficulty	ID	Video	Difficulty	ID	Video	Difficulty
1	1-f-JY-1	0.52	31	1-f-BS-3	0.35	61	1-f-BS-3	0.53
2	1-f-JY-5	0.40	32	1-f-FN-1	0.53	62	1-f-KJ-2	0.43
3	1-f-KJ-1	0.25	33	1-f-JY-1	0.41	63	1-f-JY-4	0.34
4	1-f-KJ-4	0.61	34	1-f-JY-3	0.87	64	3-f-BS-3	0.52
5	1-f-KL-2	0.57	35	1-f-JY-4	0.43	65	1-f-YW-5	0.39
6	1-f-KL-4	0.48	36	1-f-JY-5	0.44	66	1-m-KJ-1	0.63
7	1-f-KL-5	0.50	37	1-f-KJ-2	0.56	67	1-m-KJ-2	0.53
8	1-f-YW-1	0.53	38	1-f-KJ-3	0.67	68	1-m-KL-1	0.38
9	1-m-FN-2	0.48	39	2-m-FN-1	0.55	69	3-f-BS-1	0.43
10	1-m-KJ-1	0.28	40	1-f-YW-2	0.45	70	2-f-BS-2	0.80
11	1-m-YW-1	0.45	41	1-f-YW-5	0.45	71	2-f-FN-4	0.20
12	2-f-FN-3	0.59	42	1-m-BS-5	0.95	72	2-f-JY-1	0.81
13	2-f-JY-5	0.57	43	1-m-YW-5	0.52	73	2-f-JY-5	0.66
14	2-f-KL-4	0.50	44	1-m-KL-1	0.85	74	2-f-KL-5	0.46
15	2-m-BS-2	0.72	45	1-m-KL-3	0.77	75	2-f-KJ-1	0.54
16	2-m-FN-1	0.46	46	1-m-KJ-1	0.58	76	2-m-BS-3	0.34
17	2-m-JY-5	0.37	47	2-f-FN-2	0.41	77	2-m-JY-3	0.75
18	2-m-KJ-1	0.51	48	2-f-KJ-2	0.75	78	3-m-BS-2	0.57
19	2-m-KL-2	0.83	49	2-f-YW-4	0.48	79	3-f-FN-4	0.66
20	3-f-FN-2	0.45	50	2-f-FN-3	0.53	80	3-f-JY-3	0.43
21	3-f-JY-1	0.73	51	2-m-BS-2	0.70	81	3-f-KL-1	0.24
22	3-f-JY-3	0.66	52	2-m-BS-3	0.68	82	3-m-BS-3	0.39
23	3-f-KL-3	0.81	53	2-f-KJ-1	0.53	83	3-m-BS-4	0.47
24	3-m-FN-3	0.58	54	2-m-FN-3	0.42	84	3-m-FN-1	0.58
25	3-m-JY-3	0.46	55	3-m-BS-4	0.35	85	3-m-JY-1	0.58
26	3-m-KL-1	0.69	56	3-m-FN-2	0.41	86	3-m-JY-2	0.73
27	3-m-KL-5	0.91	57	3-m-FN-3	0.55	87	3-m-KJ-1	0.50
28	1-f-JY-3	0.68	58	3-m-JY-1	0.63	88	1-f-KL-1	0.58
29	3-m-BS-4	0.56	59	3-m-JY-3	0.60	89	3-m-KL-5	0.91
30	3-m-YW-5	0.37	60	3-m-KJ-2	0.42	90	3-m-YW-4	0.37

Table S2. Properties of the initial test videos

Response Category							
Condition	All	Disgust	Happy	Fear	Sad	Surprise	Angry
WQYS	30	4	9	3	2	6	6
WSJY	30	8	3	7	3	2	7
YSJY	30	5	2	5	9	8	1
All	90	17	14	15	14	16	14

Target Category							
Condition	All	Disgust	Happy	Fear	Sad	Surprise	Angry
WQYS	30	3	8	4	2	8	5
WSJY	30	4	2	6	5	6	7
YSJY	30	2	S	5	8	7	3
All	90	9	15	15	15	21	15

Target Intensity						
Condition	All	Level1	Level2	Level3	Level4	Level5
WQYS	30	9	5	6	4	6
WSJY	30	7	7	9	3	4
YSJY	30	11	S	5	5	4
All	90	27	17	20	12	14

Figure S1. Distribution, range, mean, standard deviation, and normality test of item difficulty

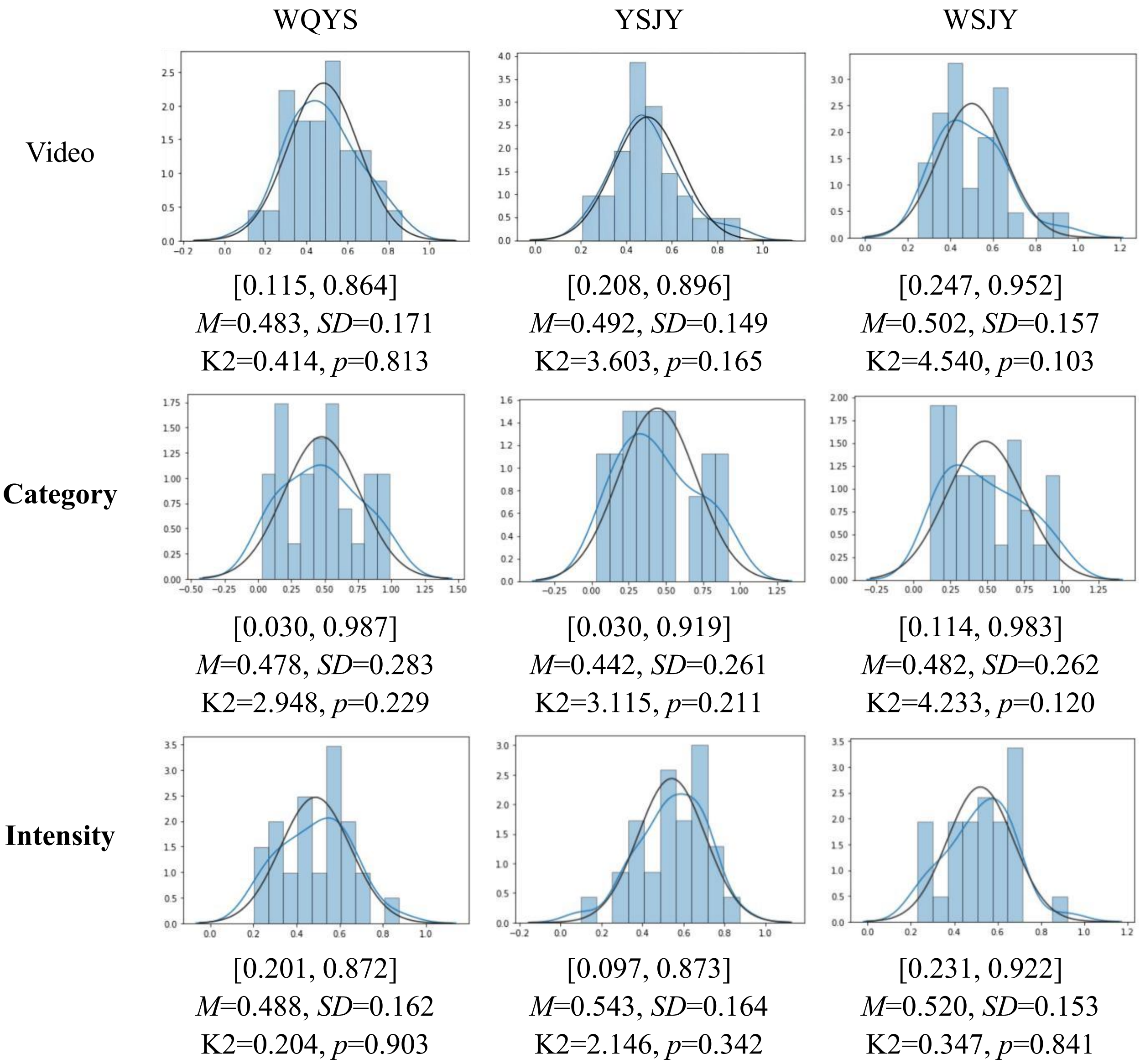

Figure S2. Distribution of Participants' Emotion Category Scores and Emotion Intensity Scores

Category

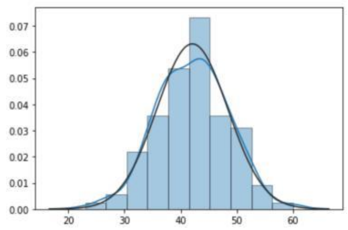

[23, 60]

M=42.09, *SD*=6.32

K2=0.092, *p*=0.955

Intensity

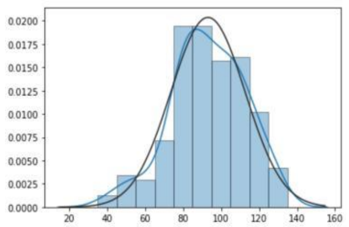

[35, 135]

M=93.11, *SD*=19.59

K2=3.547, *p*=0.146

Table S3. Correlation between Emotion Category Scores and Emotion Intensity Scores

		1	2	3	4	5	6	7	8
1	WQYS-emo	1							
2	WSJY-emo	0.293 **	1						
3	YSJY-emo	0.335 **	0.245 **	1					
4	WQYS-value	-0.090	0.049	0.042	1				
5	WSJY-value	0.03	0.099	0.134 *	0.619 **	1			
6	YSJY-value	-0.018	0.107	0.069	0.694 **	0.674 **	1		
7	EMO	0.747 **	0.699 **	0.732 **	0.001	0.121	0.072	1	
8	VALUE	-0.026	0.097	0.096	0.868 **	0.885 **	0.886 **	0.077	1

Table S4. Difficulty of the Optimized Test Videos

WQYS			WSJY			YSJY		
ID	Video	Difficulty	ID	Video	Difficulty	ID	Video	Difficulty
1	1-f-JY-1	0.30	31	1-f-BS-3	0.25	62	1-f-KJ-2	0.30
2	1-f-JY-5	0.32	32	1-f-FN-1	0.42	64	3-f-BS-3	0.48
3	1-f-KJ-1	0.12	33	1-f-JY-1	0.30	66	1-m-KJ-1	0.45
4	1-f-KJ-4	0.39	34	1-f-JY-3	0.81	67	1-m-KJ-2	0.42
5	1-f-KL-2	0.28	37	1-f-KJ-2	0.49	70	2-f-BS-2	0.62
6	1-f-KL-4	0.26	38	1-f-KJ-3	0.32	71	2-f-FN-4	0.21
7	1-f-KL-5	0.44	40	1-f-YW-2	0.37	72	2-f-JY-1	0.70
9	1-m-FN-2	0.47	41	1-f-YW-5	0.34	77	2-m-JY-3	0.80
12	2-f-FN-3	0.55	43	1-m-YW-5	0.63	78	3-m-BS-2	0.60
14	2-f-KL-4	0.43	44	1-m-KL-1	0.63	79	3-f-FN-4	0.65
15	2-m-BS-2	0.70	45	1-m-KL-3	0.62	81	3-f-KL-1	0.27
18	2-m-KJ-1	0.61	46	1-m-KJ-1	0.54	82	3-m-BS-3	0.35
19	2-m-KL-2	0.77	48	2-f-KJ-2	0.65	83	3-m-BS-4	0.44
20	3-f-FN-2	0.34	49	2-f-YW-4	0.46	84	3-m-FN-1	0.49
21	3-f-JY-1	0.68	52	2-m-BS-3	0.59	85	3-m-JY-1	0.53
22	3-f-JY-3	0.49	53	2-f-KJ-1	0.57	86	3-m-JY-2	0.64
23	3-f-KL-3	0.77	54	2-m-FN-3	0.42	87	3-m-KJ-1	0.44
24	3-m-FN-3	0.57	55	3-m-BS-4	0.29			
26	3-m-KL-1	0.68	56	3-m-FN-2	0.38			
28	1-f-JY-3	0.50	58	3-m-JY-1	0.61			
29	3-m-BS-4	0.52	59	3-m-JY-3	0.62			
30	3-m-YW-5	0.38	60	3-m-KJ-2	0.43			
